# A comprehensive characterisation of the metabolic profile of varicose veins; implications in elaborating plausible cellular pathways for disease pathogenesis

**DOI:** 10.1038/s41598-017-02529-y

**Published:** 2017-06-07

**Authors:** Muzaffar A. Anwar, Kyrillos N. Adesina-Georgiadis, K. Spagou, P. A. Vorkas, J. V. Li, Joseph Shalhoub, Elaine Holmes, Alun H. Davies

**Affiliations:** 1 Academic Section of Vascular Surgery, Imperial College, London, UK; 2 Division of Computational and Systems Medicine, Department of Surgery and Cancer Imperial College, London, UK

## Abstract

Metabolic phenotypes reflect both the genetic and environmental factors which contribute to the development of varicose veins (VV). This study utilises analytical techniques to provide a comprehensive metabolic picture of VV disease, with the aim of identifying putative cellular pathways of disease pathogenesis. VV (n = 80) and non-VV (n = 35) aqueous and lipid metabolite extracts were analysed using 600 MHz ^1^H Nuclear Magnetic Resonance spectroscopy and Ultra-Performance Liquid Chromatography Mass Spectrometry. A subset of tissue samples (8 subjects and 8 controls) were analysed for microRNA expression and the data analysed with mirBase (www.mirbase.org). Using Multivariate statistical analysis, Ingenuity pathway analysis software, DIANALAB database and published literature, the association of significant metabolites with relevant cellular pathways were understood. Higher concentrations of glutamate, taurine, myo-inositol, creatine and inosine were present in aqueous extracts and phosphatidylcholine, phosphatidylethanolamine and sphingomyelin in lipid extracts in the VV group compared with non-VV group. Out of 7 differentially expressed miRNAs, spearman correlation testing highlighted correlation of hsa-miR-642a-3p, hsa-miR-4459 and hsa-miR-135a-3p expression with inosine in the vein tissue, while miR-216a-5p, conversely, was correlated with phosphatidylcholine and phosphatidylethanolamine. Pathway analysis revealed an association of phosphatidylcholine and sphingomyelin with inflammation and myo-inositol with cellular proliferation.

## Introduction

Primary varicose vein disease, a vascular condition characterised by elongated, dilated and tortuous veins in the lower limbs, remains one of the most commonly reported chronic conditions in western medicine. While recent studies have identified familial clustering of disease, and a genetic component is generally accepted, age, occupation, pregnancy and obesity are still key risk factors. The determination of the existence of mechanistically different varicose vein “sub-phenotypes” would be important as this could reflect the different effects that various environmental factors have on disease progression. The morphological characteristics of the disease have been well documented, and known histological changes in the dilated vein include intimal hyperplasia and disruption of smooth muscle cells and the extracellular matrix^1^. However, how disease variants of risk loci disrupt the biochemistry of the cell and how these events translate into the formation of the varicose vein is still not known. Understanding how biochemical aberrations modulate metabolic pathways, enzymatic activity and normal cellular function is the natural next step in the investigation of the disease.

Metabonomics employs established and reproducible tools for the identification of the metabolic status of an individual and are highly appropriate for the evaluation of the biochemical components of a disease phenotype^2, 3^. The metabolic profiles of human varicose veins have been previously shown to be different from healthy vein tissue, when analysed using magic angle spinning (MAS) ^1^H- nuclear magnetic resonance (NMR) spectroscopy. ^1^H-MAS NMR spectroscopy allows for the profiling of intact whole tissue without the need for chemical extraction, and also provides information on metabolite mobility and metabolite localisation within the tissue^4^. Lactate, *myo-*inositol and creatine were found to be the differential biomarkers that distinguished between the healthy and diseased vein groups, and triglyceride moieties were also observed to be lower in concentration in varicose tissue^5^. However, the study in question only utilised one profiling technique and a very small sample size. Therefore, to be able to attain a more comprehensive metabolic picture, and move beyond phenotypic observation to identification of potential mechanisms, analysis of extracted metabolites from a larger number of participants is required. In addition to using multiple analytical platforms such as NMR and ultra performance liquid chromatography coupled to -mass spectrometry (UPLC-MS), microRNAs (miRNAs) have been profiled for a subset of patients to explore modulated pathways. miRNAs are small 22 nucleotide RNAs, that play an important role in cell regulation by silencing genes through targeting mRNAs for cleavage. miRNAs are involved in the regulation of many key cellular pathways and as such have been identified as potential markers for patient stratification and as therapeutic targets. Additionally, UPLC-MS, an inherently more sensitive analytical technology, can complement NMR analysis and provide further information of the perturbation of low concentration metabolites, and extend profiling coverage. This study uses three analytical techniques including NMR, UPLC-MS and miRNAs to provide a comprehensive metabolic picture of varicose vein disease, aiming to synthesise a metabolic map of the disease and identify putative cellular pathways of disease pathogenesis. Aqueous extracts from humans’ varicose vein samples and humans’ non-varicose vein control were analysed using NMR and UPLC-MS HILIC profiling. Lipid extracts from the diseased and controls veins samples were analysed using UPLC-MS. miRNAs profiling was performed on randomly selected diseases and long saphenous vein controls samples.

## Results

### Multivariate analysis of ^1^H NMR spectroscopic based profiling of vein tissue

Initial principal component (PC) modelling of the NMR data showed some stratification in the data (Supplementary Fig. 1) prompting further analysis with supervised methods.

**Figure 1:**
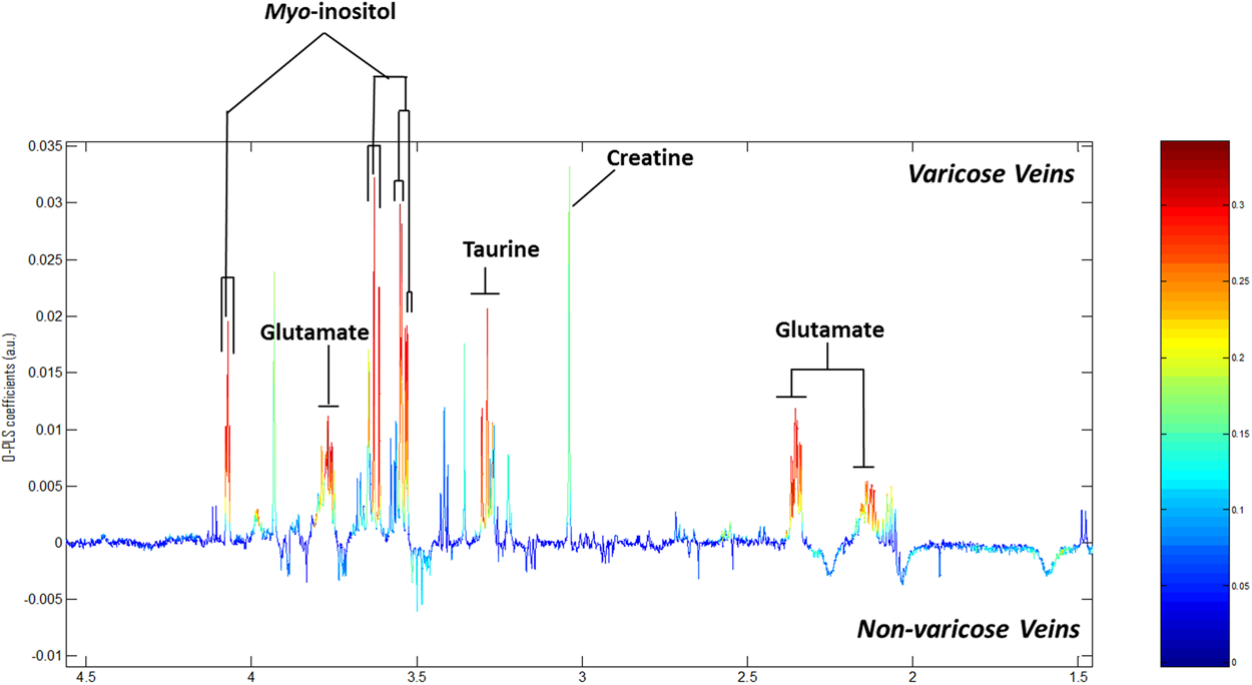
OPLS models for aqueous metabolites analysed by ^1^H NMR spectroscopy. Model is showing the differences between the varicose vein and non-varicose veins groups.

**Figure 2:**
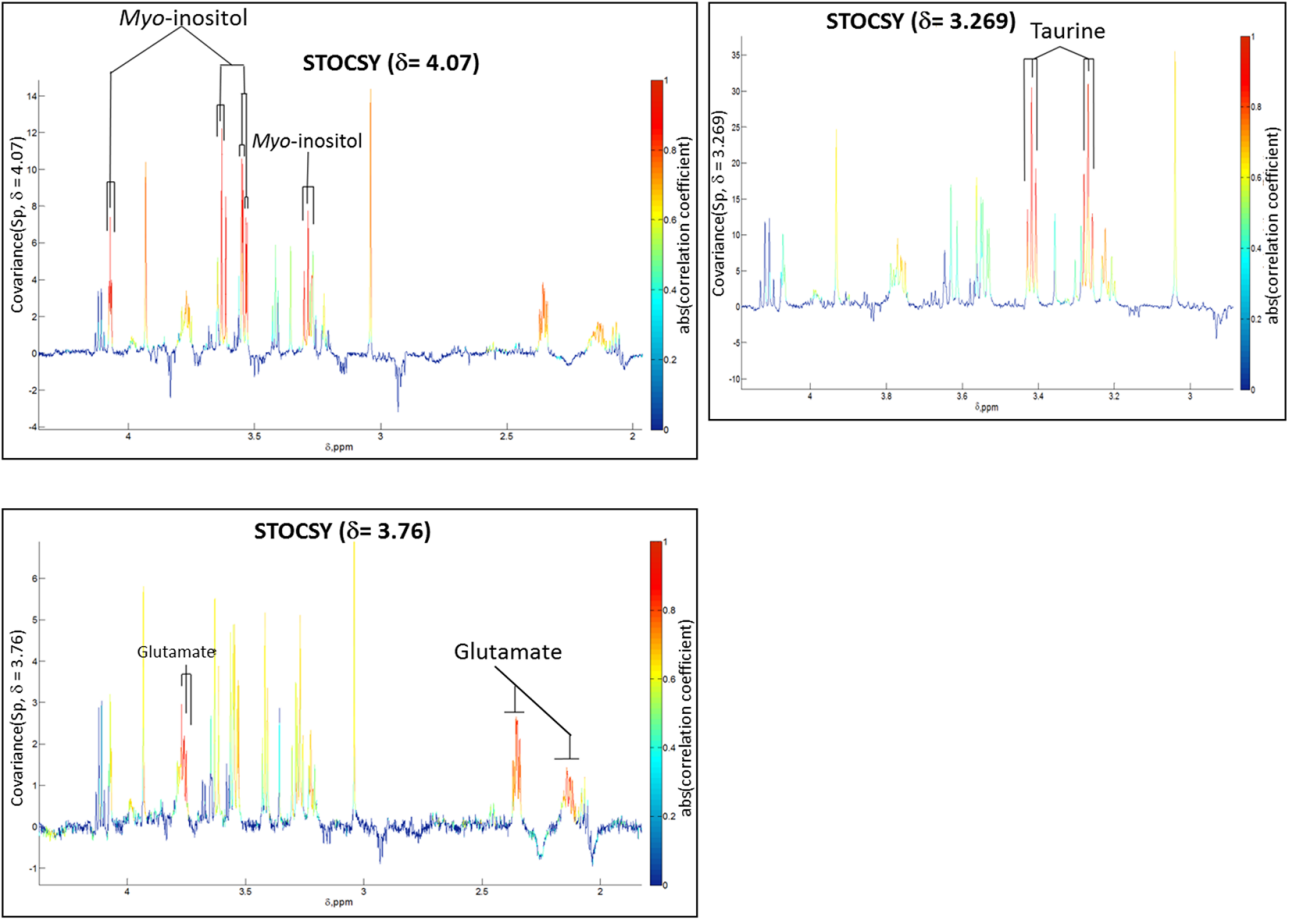
Statistical Total Correlation Spectroscopy (STOCSY). STOCSY showing intra-molecular correlation of peaks related to metabolites (taurine, *myo*-inositol and glutamate) associated with differentiation of disease from controls.

The OPLS-DA models produced with the aqueous extract ^1^H NMR data showed clear metabolic differences between the diseased and control groups. The Q^2^Y and R^2^Y values were 0.379 and 0.202, respectively, and the test statistics were validated using permutation testing (p < 0.05). The significant peaks highlighted as driving the group differences corresponded to signatures from *myo*-inositol, taurine and glutamic acid (glutamate) which were higher in the disease group (Fig. 1). These metabolites were also found to be highly significant based on the univariate statistical analysis, after correction for multiple testing (Benjamini-Yekutieli correction) with corrected *p*-values < 0.0001 for each differential metabolite. In order to identify all the correlated peaks pertaining to a particular molecule, Statistical Total Correlation Spectroscopy (STOCSY) was carried out using a selected discriminatory peak as a driver for calculation of the correlation matrix^6^. Figure 2 shows the STOCSY output of glutamate (δ3.770), taurine (δ3.269), and *myo*-inositol (δ4.07), although at δ3.269, the taurine peaks and *myo-*inositol peaks are overlapping.

### Multivariate analysis of UPLC-MS based lipid and HILIC profiling

**Figure 3:**
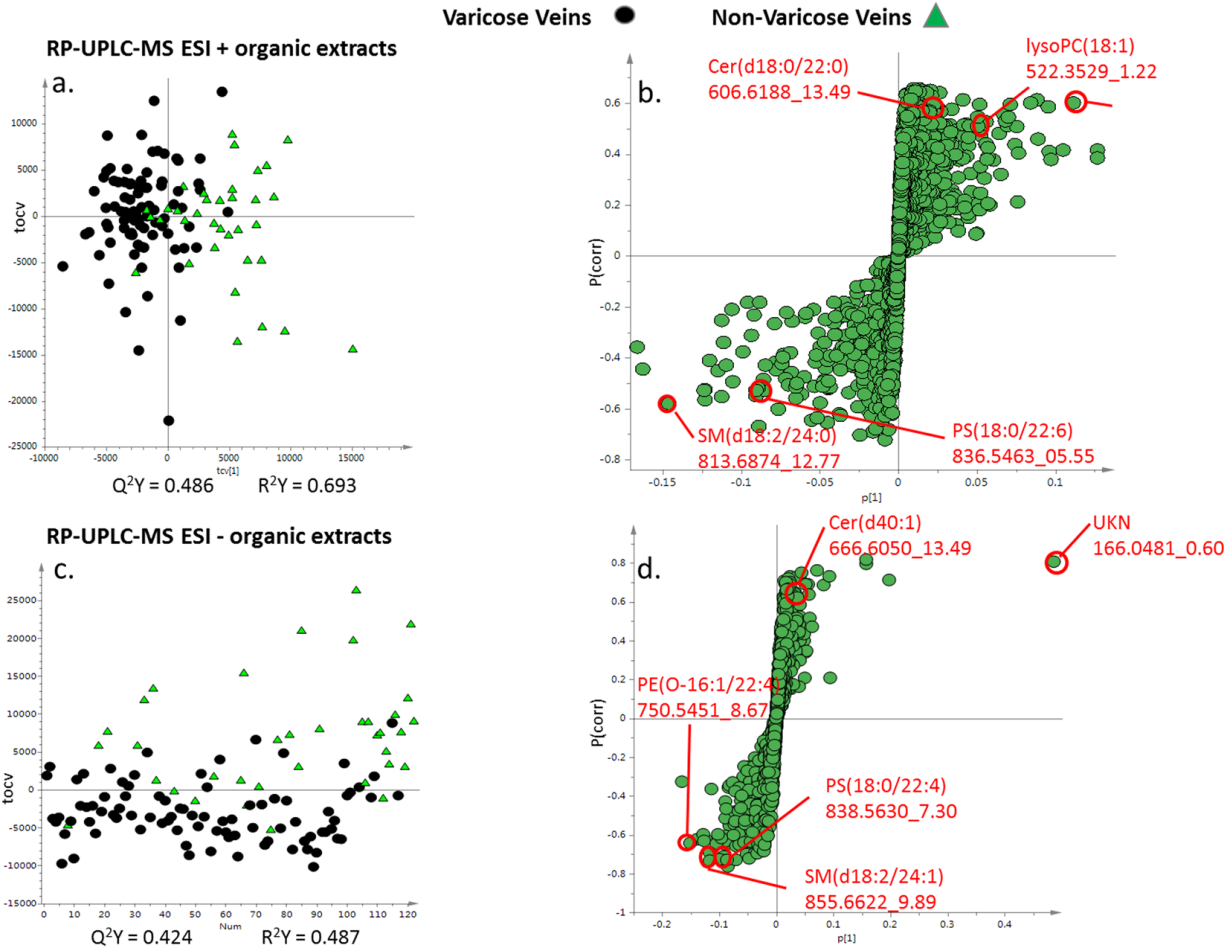
Multivariate OPLS –DA cross-validated scores and loadings S-plots for UPLC-MS based lipid profiling (ESI +/−) (**a**) and (**c**) comparing the varicose veins and non-varicose veins samples and (**b**) and (**d**) showing the S loadings plots for the OPLS-DA models. Metabolites at the outer most edges of the S plot are present in higher concentration in one or other group. Robustness of the OPLS-DA model was confirmed by permutations testing. TG Triglyceride, PC Phosphatidylcholine, SM Sphingomyelin, PE Phosphatidylethanolamine, PS phosphatidylserine.

Good instrumental stability during the lipid profiling (organic extracts) experimental run was indicated by the tight clustering of the QC samples near the middle of statistical space in the PCA scores plot from the multivariate analysis of both the electrospray ionization (ESI)+ and ESI- UPLC-MS data (Supplementary Figure 2). For the ESI+ UPLC-MS dataset, comparisons of the control and test group show some spatial trends. Subsequent orthogonal projection to latent structures discriminant analysis (OPLS-DA) modelling, after cross validation, showed a strong separation between the two groups, although there was some overlap, with the test statistics being Q^2^Y = 0.486 and R^2^Y = 0.693 (Fig. 3a). The loadings S- plot was produced for the model indicating which variables are correlating for each group. Variables clustered towards the bottom left of loadings S- plot are variables which represent metabolites more abundant in the disease group, whereas variables in the top right are down regulated in the disease group. The loadings S-plot identified triglycerides and ceramides as higher in the control group, while phosphatidylserines, phosphatidylcholines, sphingomyelins, and phosphatidylethanolamines were elevated in varicose veins group (Fig. 3b and Table 1). Details of these features are described in Table 1.

**Table 1:** Details of differential features identified between varicose and non-varicose veins groups on UP-LCMS ESI +/− and UP-LCMS HILIC +/− ESI analysis.

Met Name	Mol Formula as Detected	Retention Time (min)	m/z (found)	m/z (theor)	Fold change	p-value	Δppm	Higher in group
**UP-LCMS ESI +/− (Organic extracts)**								
Cer(d18:0/22:0)	C40H80NO2+[M+H−H2O]+	13.49	606.6188	606.6189	−2.1	0.01	0	Non-VVs
lysoPC(18:1)	C26H53NO7P+[M+H]+	01.22	0522.3529	522.3554	−1.7	0.03	−5	Non-VVs
TG(12:0/14:0/18:1) TG(12:0/16:0/16:1)	C47H92NO6+[M+NH4]+	14.77	0766.6937	766.6919	−2.1	0.03	2	Non-VVs
TG(12:0/16:0/18:1) TG(14:0/16:1/16:0)	C49H96NO6+[M+NH4]+	15.10	0794.7231	794.7232	−1.8	0.007	0	Non-VVs
TG(14:0/16:0/18:1)	C51H100NO6+[M+NH4]+	15.42	0822.7546	822.7545	−1.6	0.002	0	Non-VVs
TG(15:0/16:0/18:1) TG(14:0/17:0/18:1)*	C52H102NO6+[M+NH4]+	15.55	0837.7767	837.7741	−1.5	0.02	3	Non-VVs
TG(16:0/16:0/18:1)*	C53H104NO6+[M+NH4]+	15.71	0851.7901	851.7897	−1.5	0.01	0	Non-VVs
PC(18:0/20:3)*	C46H87NO8P+[M+H]+	08.61	0813.6229	813.6203	1.5	0.007	3	VVs
PC(38:4)*	C46H85NO8P+[M+H]+	07.06	0811.6074	811.6047	1.4	<0.0001	3	VVs
PE(O-16:1/22:6)	C43H75NO7P+[M+H]+	06.49	0748.5290	748.5276	1.4	0.01	2	VVs
PS(16:0/22:6)	C44H75NO10P+[M+H]+	04.29	0808.5168	808.5123	1.6	0.0002	6	VVs
PS(18:0/20:1)	C44H85NO10P+[M+H]+	10.07	0818.5855	818.5906	1.5	0.0002	−6	VVs
PS(18:0/20:3)	C44H81NO10P+[M+H]+	06.65	0814.5615	814.5593	1.4	0.01	3	VVs
PS(18:0/22:6)	C46H79NO10P+[M+H]+	05.55	0836.5463	836.5436	1.7	0.003	3	VVs
SM(d18:2/24:0)	C47H94N2O6P+[M+H]+	12.77	0813.6874	813.6844	1.3	0.0007	4	VVs
SM(d18:2/24:1)	C47H91N2O6PNa+[M+Na]+	09.85	0833.6557	833.6507	1.3	0.0008	6	VVs
Cer(d40:1)	C41H80NO5 [M+FA−H]−	13.49	0666.6050	666.6042	−1.2	0.01	1	Non-VVs
Alanine	C3H6NO2−[M−H]−	00.54	0088.0398	88.0404	1.9	<0.0001	−7	VVs
Creatine	C4H8N3O2−[M−H]−	00.54	0130.0611	130.0622	1.6	0.003	−8	VVs
Glutamine	C5H8NO4−[M−H]−	00.54	0146.0459	146.0459	1.8	0.0002	0	VVs
Guanosine	C10H12N5O5−[M−H]−	00.54	0282.0840	282.0844	1.4	0.005	−1	VVs
Inosine	C10H11N4O5−[M−H]−	00.54	0267.0724	267.0735	1.6	0.001	−4	VVs
PC(18:0/20:3)	C47H87NO10P− [M+FA−H]−	08.62	0856.6094	856.6073	1.4	0.008	2	VVs
PE(O-16:1/22:4)	C43H77NO7P−[M−H]−	08.67	0750.5451	750.5443	1.2	0.004	1	VVs
PE(O-16:1/22:6)	C43H73NO7P−[M−H]−	06.53	0746.5136	746.513	1.3	0.007	1	VVs
PI(18:2/18:0)	C45H82O13P−[M−H]−	06.01	0861.5527	861.5499	1.4	<0.0001	3	VVs
PI(36:1)	C45H84O13P−[M−H]−	07.40	0863.5676	863.5655	1.4	0.001	2	VVs
PS(16:0/22:6)	C44H73NO10P−[M−H]−	04.33	0806.4999	806.4978	1.6	0.0005	3	VVs
PS(18:0/20:1)	C44H83NO10P−[M−H]−	10.15	0816.5787	816.576	1.4	0.01	3	VVs
PS(18:0/20:3)	C44H79NO10P−[M−H]−	06.72	0812.5485	812.5447	1.4	0.004	5	VVs
PS(18:0/22:4)	C46H81NO10P−[M−H]−	07.30	0838.5630	838.5604	1.5	0.0006	3	VVs
PS(18:0/22:6)	C46H77NO10P−[M−H]−	05.60	0834.5309	834.529	1.4	0.0007	2	VVs
SM(d18:2/24:0)*	C47H93N2O6P−[M+FA−H]−	12.79	0858.6876	858.6781	1.3	0.0005	11	VVs
SM(d18:2/24:1)	C48H92N2O8P−[M+FA−H]−	09.89	0855.6622	855.6597	1.4	<0.0001	3	VVs
Uridine	C9H11N2O6−[M−H]−	00.55	0243.0616	243.0623	1.4	0.006	−3	VVs
**UP-LCMS HILIC +/− Analysis (Aqueous extracts)**								
Creatine	C4H10N3O2+[M+H]+	6.623	132.0773	132.0767	1.3	<0.0001	4.5	VVs
Inosine	C5H5N4O+[M+H]+	6.073	137.0463	137.0457	1.3	0.007	4.3	VVs
PC (16:0/20:4)	C44H81NO8P+[M+H]+	4.737	782.5684	782.5694	1.4	0.009	−1.2	VVs
Glutamate	C5H8NO4−[M−H]−	7.467	146.0455	146.0458	1.5	<0.0001	−2	VVs
Inosine	C10H11N4O5−[M−H]−	3.167	267.0729	267.0734	1.24	0.01	−1.8	VVs
Taurine	C2H6NO3S−[M−H]−	5.808	124.0071	124.0073	1.17	0.07	−1.6	VVs
Uridine	C9H11N2O6−[M−H]−	1.517	243.0617	243.0622	1.2	0.04	−2	VVs

TG Triglyceride, PC Phosphatidylcholine, SM Sphingomyelin, PE Phosphatidylethanolamine, PS phosphatidylserine, PI phosphatidylinositols

Corrected *p*-values using Benjamini-Yakutieli multiple testing corrections. *Tentative assignments.

Similar distribution trends were also seen in the ESI- UPLC-MS dataset with the positions of the control and disease subjects within the model, with some separation between the classes on PC1 (Supplementary Figure 2). Further supervised analysis with OPLS-DA modelling show a better separation, with good test statistic values (Q^2^Y = 0.424 and R^2^Y = 0.487). The metabolites identified as driving the class differences, again using the loadings S-plot, included phosphatidylserines, phosphatidylcholines, sphingomyelins, ceramides, phosphatidylethanolamines and phosphatidylinositols (Fig. 3c and d, and Table 2). Additionally, several other small molecules demonstrated high correlations with the disease or control group and exhibited retention times at or close to the solvent-front. These were inosine, taurine, guanosine and uridine, which were elevated in patients with varicose veins. Furthermore, there were some differential unknown metabolites observed as well on UPLC MS ESI+/− modes. Details of these are listed in Supplementary Table 1. Future work may be required to identify these unknown metabolites and their relevance in veins.

**Table 2:** Details of human vein tissue and Demographic details of patients.

	Non-varicose veins	Varicose veins	P value
	*n* = *35*	*n* = *80*	*—*
Type of vein tissue	Cephalic = 3	Truncal = 44	*—*
	Long saphenous = 15	Varicosities = 36	
	Inferior epigastric/mesenteric = 8		
	Facial = 9		
CEAP clinical score	*—*	CEAP 2 = 4	*—*
		CEAP 3 = 66	
		CEAP 4 = 9	
		CEAP 5 = 1	
Recurrent varicose veins	*—*	9	*—*
Sex	Female = 6	Female = 40	*—*
	Male = 29	Male = 40	
Age, years	32–85 (mean 62.7)	18–82 (Mean 45.16)	<0.0001
HTN	21	14	<0.0001
Diabetes mellitus	9	2	0.002
PAD	12	8	0.02
IHD	12	2	0.001
Stroke	6	2	0.03
Cancer	1	0	0.3
Connective tissue disorders	0	0	*—*
Aspirin	18	12	0.0002
Simvastatin	20	12	<0.0001
Lisinopril	17	5	<0.0001
Amlodipine	6	5	0.18

CEAP Clinical Aetiological Anatomical Pathophysiological. IHD ischemic heart disease, HTN hypertension, PAD peripheral arterial disease.

**Figure 4:**
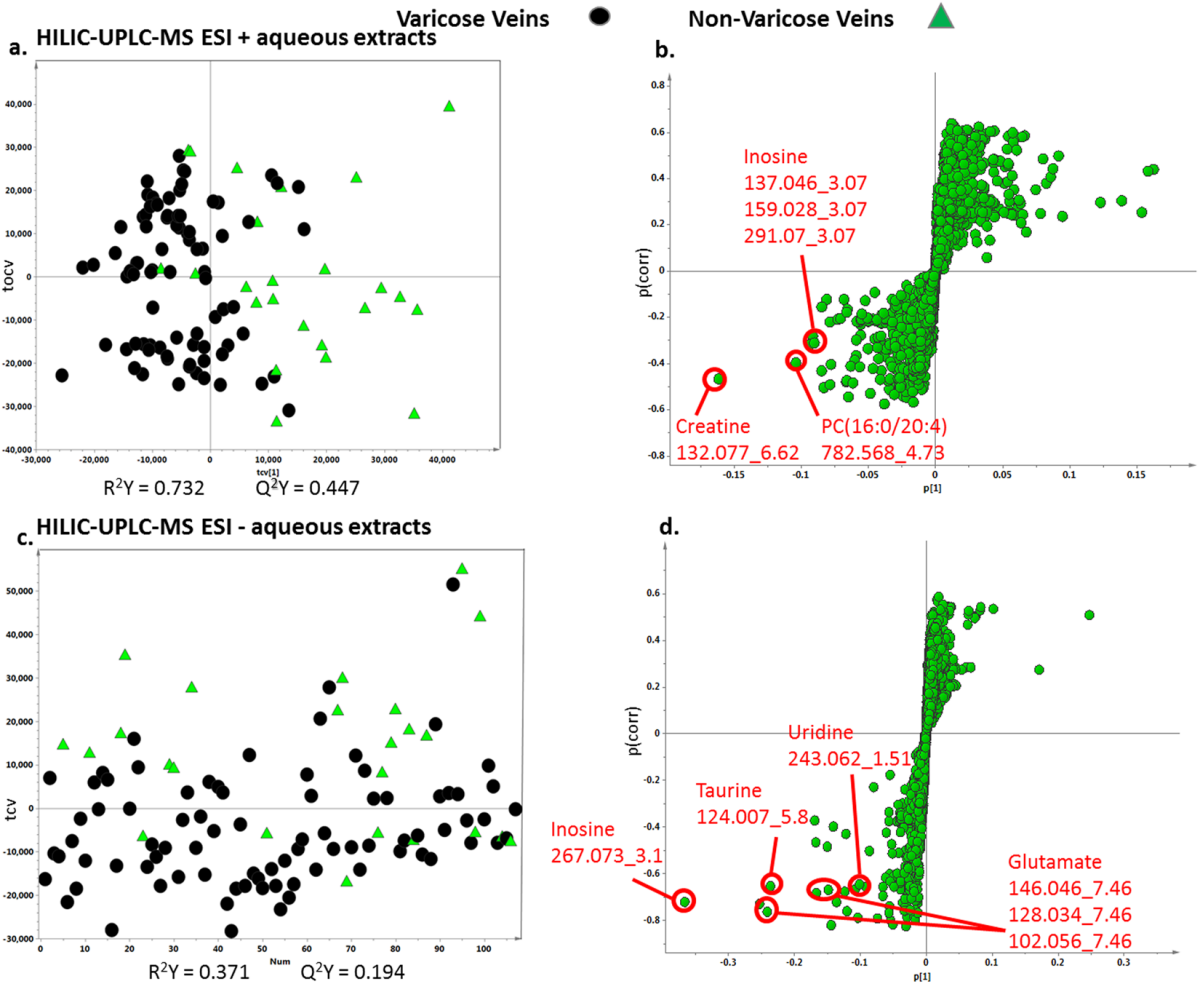
Multivariate OPLS –DA cross-validated scores and loadings S-plots for UPLC-MS HILIC based profiling (ESI +/−). (**a**) and (**c**) Comparing the varicose veins and non-varicose veins samples. (**b**) and (**d**) Showing the S loadings plots for the OPLS-DA models. Metabolites at the outer most edges of the S plot are present in higher concentration in one or other group. Robustness of the OPLS-DA model was confirmed by permutations testing. PC Phosphatidylcholine.

Principal component analysis of the aqueous extracts by UPLC-MS HILIC ESI+ also indicated that there were differences between the classes (Supplementary Figure 2). There was some separation of groups on PCA score plots with clustering of samples in varicose veins group. Therefore cross-validated OPLS-DA models were again run to identify differences between the groups (Fig. 4a). These identified inosine, creatine and phosphatidylcholine (PC) which were higher in varicose veins group (Fig. 4b). The same process was also repeated for the aqueous extracts analysed on UPLC-MS HILIC ESI-, and both the PCA analysis (Supplementary Figure 2) and the OPLS-DA modelling showed clear separation of varicose and non-varicose veins samples (Fig. 4c). Further interrogation of the loadings S-plot identified inosine, glutamate, uridine and taurine as the differential metabolites, and these were present in higher concentration in varicose veins group relative to non-varicose veins samples (Fig. 4d). Details of differentials features identified in HILIC+/− ESI are shown in Table 1.

Patients participating in the test group, attending hospital for varicose vein intervention, were on average younger and with fewer co-morbid conditions than the controls; furthermore many were taking medications, such as aspirin, simvastatin and lisinopril, at the time of sample collection (Table 2). Multivariate partial least square (PLS) regression modelling approach showed that none of these secondary factors produced any significant model. The sole exception was a PLS-DA model for hypertension in UPLC-MS lipid profiling in negative ionisation, whereby the R^2^Y and the Q^2^Y for that model were 0.207 and 0.053 on first component with the p- value 0.05 for the first component. However, when the second orthogonal component was added to the model the Q^2^Y value was reduced to a negative value and p value changed to 1. This would indicate that the initial model was “over-fitted” and thus it is unlikely hypertension was a real bias which would skew the main comparisons (Supplementary Table 2).

The PCA scores plots for all the datasets showed some dispersal within the control group itself, therefore to evaluate if these within-group variations would affect the main models, PCA models containing only control samples were constructed, and the scores plots were coloured according to vein origin (Supplementary Figure 3). This highlighted that there were no significant metabolic differences between control veins of different anatomical locations. Furthermore, when models comparing the disease group samples with only control veins from the long saphenous vein (the most common control vein location) were created similar results to the main models were produced, albeit with lower significance values, which is to be expected when reducing sample size (Supplementary Figures 4, 5 and 6).

### miRNA analysis and Pathway analysis

This experiment highlighted differential expression of 7 miRNAs in varicose vein disease which are listed in Table 3, together with their expected regulated pathways. The elevated levels of hsa-miR-4459, hsa-miR-135a-3p and hsa-miR-216a-5p in varicose vein tissue are of particular interest as they regulate signal cascades that directly influence cell proliferation and survival.

**Table 3:** Differentially expressed miRNAs in the varicose vein cells, together with the fold change ratio, overexpression analysis p-value and the relevant target processes which each miRNA regulates.

miRNA	Fold change	P value	Target Genes	Target Processes
hsa-miR-642a-3p	−2.913896	4.14 × 10^−3^	285	Biological regulation, transcription, nucleobase-containing compound metabolism, fatty acid metabolism
hsa-miR-718	−2.582323	—	11	Pentose Phosphate pathway
hsa-miR-4459	−2.5259194	2.72 × 10^−2^	349	EGF receptor signalling pathway, primary and secondary bile acid synthesis
hsa-miR-135a-3p	−2.2473247	1.02 × 10^−3^	718	PI3K pathway, EGF receptor pathway, TGF-beta signalling pathway, Thiamine metabolism
hsa-miR-363-3p	−2.2425704	1.5 × 10^−3^	893	GRH receptor pathway, Leucine and Isoleucine biosynthesis
hsa-miR-216a-5p	8.4616585	8.79 × 10^−3^	287	Wnt signalling pathway, TGF signalling pathway
hsa-miR-136-5p	4.2504244	—	270	glycosaminoglycan biosynthesis

**Figure 5:**
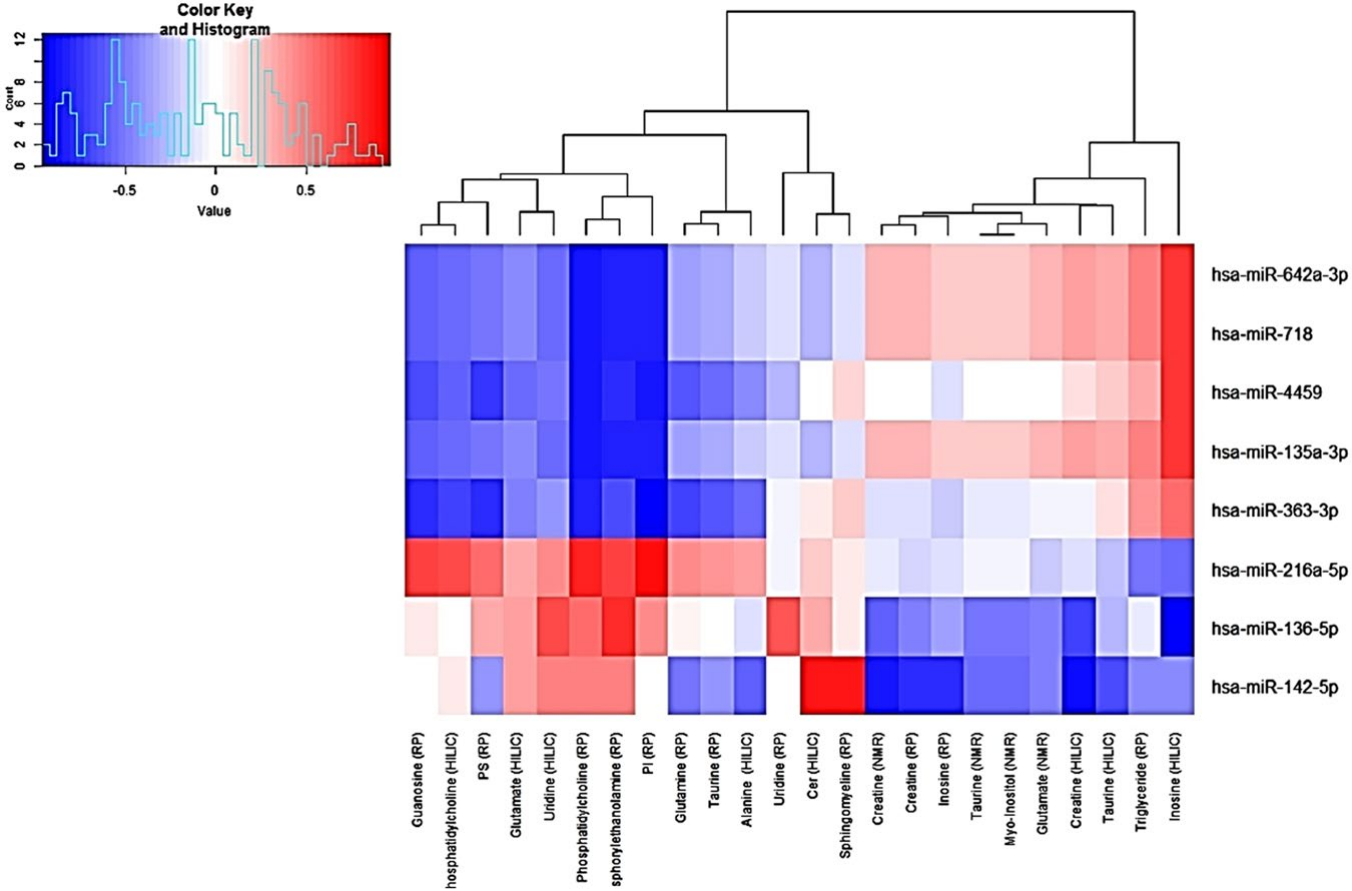
Correlation heatmap and a dendrogram. A correlation heatmap and a dendrogram of the spearman correlation mapping between the significant metabolites (across the bottom) and the differentially expressed miRNAs (along the right). Areas of intense red colour signify a high level of correlation between the expression of that miRNA and levels of the matching metabolite, whereas blue areas signify anti-correlation. The dendrogram at the top shows correlation distances by clustering the metabolites hierarchically.

**Figure 6:**
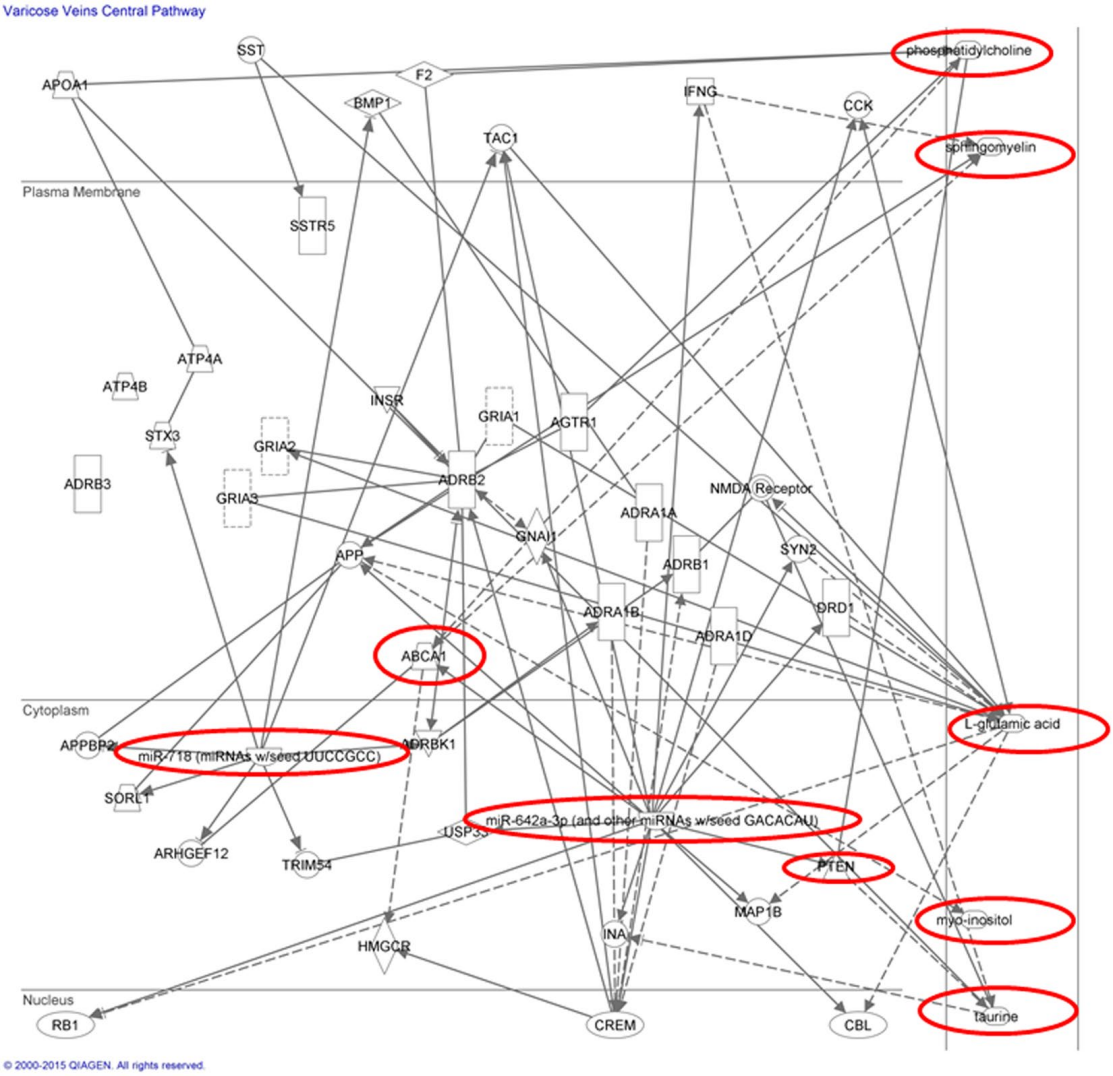
Ingenuity Pathway Analysis showing a network biology system for the interaction between both the miRNAs and the metabolites identified from the metabonomic analysis.

A Spearman correlation matrix was constructed to investigate the correlation between miRNA expression and the levels of the significant metabolites identified through the analytical chemistry. The resultant heatmap and dendrogram are provided (Fig. 5). This highlighted that hsa-miR-642a-3p expression levels were highly correlated with levels of inosine within the vein tissue, as are expression levels of hsa-miR-4459 and hsa-miR-135a-3p. Expression of miR-216a-5p, known to regulate the Transforming Growth Factor beta (TGF-β) signalling pathway, conversely showed high levels of correlation with a variety of metabolites including phosphatidylcholine, phosphatidylethanolamine, and uridine. Finally, hsa-miR-142-5p showed a very strong correlation with sphingomyeline levels. IPA analysis included the identified miRNAs in addition to the previously identified differential metabolites. IPA provides a putative network based on statistical probability. The analysis used an IPA core analysis with a miRNA target filter, and this was continued to identify a causal network. This process is similar to gene-set enrichment, but with the additional power of incorporating cause-effect relationships. Figure 6, the output of the IPA, shows the association between the highlighted miRNAs, hsa-miR-642a-3p (representing the miRNAs with seed GACACAU) and hsa-miR-142-5p (and other miRNAs with seed UUGGCUU) and levels of sphingomyeline, phosphatidylinositol, glutamate and *myo*-inositol. The analysis revealed that the miRNAs regulate production of a variety of receptors which converge on the activation of a number of enzymes involved in phospholipid homeostasis and activation of MAP kinase. ABCA1, an ATP-binding cassette transporter involved in phospholipid homeostasis, and PTEN, a phosphatase and tensin homolog which catalyses the conversion phosphatidylinositol (3,4,5)-trisphosphate (PIP3) to phosphatidylinositol 4,5-bisphosphate (PIP2), are predicted to contribute to the regulation of both the PI3K/AKt and MAPK pathways, which are known to be involved in cell proliferation. The identities of remaining nodes of the pathway are defined in the supplementary section.

## Discussion

The aim of this study was to provide a comprehensive characterisation of the metabolic profiles of varicose and non-varicose veins, and to identify any metabolic differences between the two. Chemometric analysis of the tissue aqueous extracts revealed that the polar metabolites creatine, glutamate, *myo*-inositol, taurine and inosine were present in higher concentrations in varicose veins. The validity of these results was reenforced by the agreement of both MS-based analytical techniques and the results of the NMR spectroscopy in identification of the differential metabolites in varicose vein disease. In addition the non-polar metabolites sphingomyelin and several phospholipids were also found to be elevated in the disease group. Pathway analysis provided a potential network of molecular interactions between these metabolites and identified miRNAs within VV; this network paves the way for further investigation of disease etiology. Variations in age, sex, differences in past medical history and medications had no correlation with any metabolic differences noticed between the groups.

Glutamate, one of the key metabolites identified by the multivariate modeling, is an alpha-amino acid, a key metabolite in many aspects of cellular metabolism, and plays a central role in hepatic amino acid metabolism, glycolysis, gluconeogenesis and the tricarboxylic acid cycle^7^. In adition glutamate is also involved in the production of gamma-aminobutyric acid (GABA) in the GABA-shunt and also part of glutathione synthesis^8^. Glutathione, being one of the primary conjugative molecules of phase two detoxification, is an important factor in the cellular defence mechanism and also helps regulate cell proliferation and apoptosis^9^. However there were no discernable changes in glutathione levels detected in the samples.

Another of the significantly changed metabolites is taurine. Taurine, a ubiquitous organic osmolyte, is one of the most abundant amino acids within the body, although it is not involved in protein synthesis. Metabolically derived from cysteine, taurine is involved in the formation of bile salts, regulation of cell volume and modulation of intracellular calcium concentration, essential for the function of signal transduction pathways^10^. Administration of taurine has also been shown to protect from stress-induced vascular endothelial cell apoptosis^10, 11^, and high levels of taurine are thought to promote cell survival.

*Myo*-inositol, one of the more abundant inositol isomers, is a carbocyclic polyol and is also a structural component of a number of secondary cellular messengers such as inositol phosphates, phosphatidylinositol and phosphatidylinositol phosphate lipids. *Myo*-inositol, like taurine, predominately operates as an intercellular osmolyte, and is also involved in the Akt signalling pathway, membrane trafficking and regulating apoptosis. Cytosolic *myo*-inositol is essential for cell survival and promotes growth and proliferation^12^.

Sphingomyelin was elevated in VV tissue, and correlation analysis showed its concentration is closesly linked to the hsa-miR-142-5p part of the predicted VV network (Fig. 5). Sphingomyelin has also previously been observed in varicose veins by Tanaka *et al*. using matrix assisted laser desorption ionization imaging^13^. Sphingomyelin is the most abundant type of sphingolipid and constitutes a key structural component of cellular membranes and vesicles. Sphingomyelin acts as a platform for the regulation of transmembrane signalling, and also intracellular vesicle trafficking as bilayer sphingomyelin levels are involved in chemotaxis-based migration. This modulation extends to transferrin-mediated cell proliferation triggering cell growth and division^14^.

The statistical analysis showed that there is an increase of cellular concentrations of a wide range of phospholipids which are the key component of the lipid bilayer and are essential for the viability of eukaryotic cells. While both phosphatidylserine and phosphatidylethanolamine, have been shown to be involved in intracellular signal transduction and cell proliferation, of most interest is phosphatidylcholine. Phosphatidylcholine, a glycerophospholipid, is synthesised by the CDP-choline pathway or through the hepatic phosphatidylethanolamine methyltransferase. As a key component of membranes and lipid messengers, phosphatidylcholine is directly involved in cell cycle progression and cell proliferation^15^. Aberrant activation of phosphatidylcholine production is usually through induction of the normal PI3K/Akt pathway, and is a key feature of many forms of cancer. Furthermore, it has been shown that increased phosphatidylcholine due to the increased expression of choline kinase A (CKa) is related to hypoxia, as the gene encoding for CKa includes a hypoxia induced element in the promoter region^16^; this means that during hypoxic conditions, cells will increase choline uptake potentially triggering downstream cell cycle progression. Phosphatidylcholine is also a substrate for phospholipase A2 (PLA2). PLA2 causes hydrolysis of phosphatidylcholine into free fatty acids such as arachidonic acid (AA) and lysophosphatidylcholine. AA is a precursor molecule for prostaglandins and leukotriene, both responsible for chronic inflammation^13^. We didn’t detect phosphatidylcholine with AA incorporated, however, we detected phosphatidylcholine (20:3).

Finally the two other metabolites of note are inosine and creatine, which both follow the theme of hypoxia-induced metabolic changes; inosine is known to be released during hypoxia^17^ and increased creatine has been seen in dilated cardiomyopathy^18^. Correlation matrix analysis (Figs 5 and 6) showed that inosine levels may be highly regulated by miR-4459 and mIR-216a-5p, in addition to miR-642a-3p the key miRNA in the presented VV network, with taurine levels also showing a strong correlation with the same miRNAs predicted to be under the direct regulation of PTEN.

*Myo*-inositol, found to be elevated in varicose vein tissue, both in this study and previous work by *Anwar et al*.^5^, is also related to this process, as it constitutes the precursor molecule for phosphatidylinositol, a substrate for the PI3K signalling pathway. In fact, PI3K constitutes a key component of the suggested varicose vein pathway from the IPA (Fig. 6). Identified miRNAs mIR-216a-5p (upregulated in VVs) and miR-4459 (downregulated in VVs) are predicted to regulate expression of membrane receptors TFG-beta receptor 1 and EGF receptor, respectively. These receptors play a key role in the activity of PI3K. Furthermore expression of PI3K itself is predicted to be controlled by miR-135a-3p. The altered activies of these particular miRNAs, based upon our putative interaction network, seem to indicate that there is a phenotypic switch in the expression profile of the varicose vein cells, likely in smooth muscle cells, with dysregulation of the systems controlling cell growth and proliferation.

A similar narrative can be discerned from evaluating the results while focusing on sphingolipid metabolism. Sphingolipids share a common 18 carbon backbone, with different accessory structures giving rise to the various members of the sphingolipid family. The metabolic network could be thought of a web of interconnected pathways, branching out from a common progenitor molecule and coming together to terminate in a common catabolic end-point. The various intermediate molecules on each branch of the network, however, have highly diverse functions, and as such the metabolic trajectory each molecule is highly regulated to ensure correct cellular function. Sphyngomyelin, mentioned above and found to be raised in varicose veins, is synthesised by the transfer of a phosphocholine group from phosphatidylcholine (also shown to be elevated in varicose veins) to ceramide, by sphingomyelin synthases (SMSs)^19^; this reaction also produces diaclyglycerol. Diaclyglycerol is typically converted to phosphatidic acid by the diacylglycerol kinases; a process that has been shown to protect myocardium cells from hypertrophy when subjected to abnormally high pressure and stretch forces^20^. However, sphingomyelin is catabolised by the hydrolysis of the phosphocholine group by sphingomyelinases (SMase), the activity of which is closely linked with local pH and oxygen levels^21^; Indeed studies investigating the effect of sphingomyelinase deficiency has shown that cells that do not catabolise sphingomyelin efficiently are protected from stress-induced apoptosis^22^. Each player in this complex set of interactions has been shown to be a bioactive lipid, with sphingomyelin, phosphocholine, phosphatidylcholine, ceramide and DAG all exerting an influence on the survivability of the cell, albeit an opposing one. The first three seemingly are encouraging growth and survival, whereas the latter two promoting cell death and quiescence^23^. Taken together this could indicate that the local chemical environment and physical duress which cells are exposed to following chronic blood stasis could be triggering changes in the balance of metabolism and disrupting lipid homeostasis, leading to the production of certain pro-survival molecules with the relevant downstream effects.

This study has presented a metabolic characterisation of the VV disease, highlighting the molecular processes underlying VV pathogenesis and progression. It has been well established that such a phenotypic switch will affect vein wall contraction and result in vein wall relaxation and weakness, which may be the cause of the tortuous and loose appearance of the diseased vein. The identified metabolites suggest that the processes of cell proliferation and survival should be further investigated with a view to identifying potential therapeutic drug targets. Development of drug therapy in venous disease may help in halting the disease progression as well as a preventative measure for individuals with a genetic propensity for the development of the disease.

One of the key limitations of the work presented here is the heterogeneity of the control group. The control group did not accurately match with the varicose veins group in terms of demographic features, past medical history and the medications. Factors such as age, medications and past medical history may influence the metabolic profile of vein tissue. However, to our knowledge, there has been no demonstration of the effect of age on metabolic profile of vein tissue so far. The control group included great saphenous vein samples from patients undergoing lower limb bypass operations or amputations in addition to vein samples from other anatomical locations such as the neck and lower abdomen, which are subject to different biomechanical stresses than the GSV. While statins and most other cardiovascular medications have a short half-life (less than 24 hours)^24^ and neither parent drugs nor their metabolites were detected in our NMR or LCMS based profiling, it is possible that the above factors confounded the presented results by pharmacological effect. It is, nonetheless, important to stress that despite these issues the varicose vein samples were consistently different from the control groups in the statistical modelling, suggesting that any variability within the non-varicose veins groups was far outweighed by the differences between the two groups. Ultimately due to the ethical issues surrounding vein sample retrieval from often young and otherwise healthy individuals, it may prove challenging if not impossible to correctly age match groups in future varicose veins studies.

Another consideration for the future is CEAP classification, the scheme by which the disease is classified into different grades based on severity and type of presentation^25^, and how CEAP grading relates to metabolic phenotypes. Future studies should potentially explore any differences of metabolites in tissue, serum and urine between patients of different disease grades. This could aid clinicians in following disease progression and help tailor therapy accordingly. Varicose vein disease is a multifactorial disease influenced by both environmental factors such as occupation, obesity, pregnancy and also has a strong familial predisposition. Environmental stimuli in the patient exposes the vein wall to high pressure and hypoxia, which in combination trigger molecular changes in the vein wall leading to vein wall relaxation and dilatation. This work has shown that the metabolic profiles of human varicose veins are systematically different from non-varicose veins tissues, as well as exemplifying the utility of the metabonomic approach in understanding the molecular changes at cellular level of vascular disease.

## Methods

Ethical approval was obtained for the research from the Riverside Research Ethics Committee (RREC 3092). The research has also been registered to the Imperial College London Research Services, and the Imperial College Healthcare NHS Trust Research and Development department and all the experiments were performed in accordance with the department’s guidelines and regulations. All the specimens collected were anonymised according to the rules set out in the Human Tissue Act (2004). Informed consent was obtained from all the patients involved in the study. Varicose veins were retrieved from patients who were treated with ligation of sapheno-femoral junction and stripping of great saphenous vein (GSV) or phlebectomies within Imperial College London Healthcare NHS Trust. Initial assessment of the patient was performed in outpatient department. Duplex ultrasound imaging of the lower limb venous system was performed for all varicose veins patients by accredited vascular scientists where the presence of venous reflux was assessed. Control veins were obtained from patients undergoing any surgery which involved the routine removal of non-varicose veins that would otherwise be discarded, including residual vein following vein harvest for extremity arterial bypass. Patients with a current or past history of varicose veins were not included in the non-varicose vein control group. Varicose and non-varicose veins of the affected limbs of patients with a history of deep vein thrombosis (DVT) or superficial thrombophlebitis were excluded from the study. Patients with congenital diseases associated with varicose veins, including Klippel-Trenaunay Syndrome, arterio-venous malformations, secondary varicose veins, or human immunodeficiency virus (HIV) infection were not included in the study.

In total, 80 participants with varicose veins and 35 with non-varicose vein samples were included in the study. Tissue and demographic details are provided in Supplementary Table 2. Samples were cut and prepared on dry ice in the class II vacuum hood in the Division of Computational and Systems Biology at Imperial College London. Samples were cut circumferentially and an average sample weight of 140+/− 5 mg was used. However, some non-varicose veins were of low weight especially inferior epigastric veins retrieved from hernia surgery and facial veins retrieved from carotid endarterectomy. The effect of different sample weight was compensated by respectively suspending the metabolites in different concentrations of solvents during their extractions process, such that the tissue to solvent ratio remained constant.

### Chemicals used

The organic solvents used were chloroform, acetonitrile, DCM, ISP, hexane and MTBE (Sigma-Aldrich Gillingham, UK). Methanol (Fisher) and water were (Fluka) both LC-MS grade. Additionally, formic acid, leucine enkephalin, ammonium formate (HPLC grade) and sodium formate, were obtained from Sigma-Aldrich, Gillingham, UK. Deuteriated chloroform with 0.05% tetramethylsilane (TMS) was obtained from Goss Scientific, UK. Biological tissue was handled in Class II biological cabinet and local laboratory protocols were strictly followed. Chloroform was handled in safety cabinet due to hazardous risks involved with chloroform. Local laboratory safety precautions were followed while handling liquid nitrogen.

### Extraction of metabolites

Metabolite extraction was performed as previously optimised in human vein tissue^26^. Aqueous and organic extractions were performed sequentially on the tissue samples. Organic extraction was performed first followed by aqueous extraction^26^ (please see Supplementary Information for the details of extraction protocol).

### ^1^H-NMR spectroscopic analysis of aqueous extract

Two aliquots holding dried aqueous extracts from 80 varicose vein and 35 non-varicose vein samples were prepared for NMR spectroscopy using the method previously by Anwar *et al*.^26^ (please see Supplementary information for the details of aqueous extracts preparation). Aqueous vein extracts were analysed using ^1^H NMR spectroscopy at a field strength of 14.1 T (^1^H 600.29 MHz) using a 5 mm broadband inverse configuration probe with a z axis magnetic field-gradient capability. The spectrometer was equipped with a Bruker Sample Jet system set to 5 mm shuttle mode with a cooling rack of refrigerated tubes at 6 °C and controlled via a Bruker Avance III console (Bruker, Rheinstetten, Germany). A standard 1D pulse sequence [recycle delay (RD)-90°-t_1_-90°-t_m_-90°-acquire free induction decay (FID)) with water suppression applied during RD of 2 s and t_m_ was used to acquire data for each sample. A total of 512 scans were acquired into 64 K data points. The spectral width of δ^1^H 20.00 and acquisition time of 1.36 sec were employed for all aqueous experiments. Aqueous Spectra acquired from NMR spectroscopy were phased and calibrated using chemical shift of sodium 3-trimethylsilyl-1-[2,2,3,3,-^2^H_4_] propionate (TSP) at δ^1^H 0.00 in TOPSPIN 3.0 software (Bruker BioSpin, Rheinstetten, Germany). The baseline was corrected manually. Spectra were imported into MATLAB R2009b (Mathworks™, 2009) using an in-house algorithm built in MATLAB. The spectral region containing the water resonance (from δ^1^H 4.68 to 5.24) was removed from all spectra. In addition, the resonance of TSP (from δ^1^H −1 to 0.2) was removed from all spectra. All spectra were subsequently aligned and normalised using probabilistic quotient normalisation^27^. NMR assignments were performed by observing signal peak, chemical shift and comparing this to a list of standards available on central database HMDB, and an internal database. Statistical Total Correlation Spectroscopy (STOCSY)^6^, which computes the correlation between variables in the spectrum, by taking advantage of the multicollinear nature of NMR data, was also employed for the identification of related peaks, aiding the metabolite identification process.

### UPLC-MS analysis of aqueous and organic extracts

UPLC-MS analysis was performed with the protocols previously published by our group^26, 28^. A total of 50 µl from each sample was pooled to make a quality control (QC) sample^29^. QC samples were injected every 10 samples throughout the run to monitor the stability of the UPLC-MS instrument. Details of samples preparation for UPLC-MS analysis is given in supporting information section. Organic extracts (lipid profiling) from 80 varicose vein and 35 non-varicose vein samples were analyzed as previously described^28^ using an Acquity UPLC system (Waters Ltd Elstree, UK) coupled to a Q-TOF Premier mass spectrometer (Waters Technologies, Ltd. Manchester, UK). For chromatography of organic extracts, an Acquity UPLC column CSH (1.7 µm, 2.1 × 100 mm, Waters, USA) was used. Analysis of aqueous extracts from 80 varicose vein and 24 non-varicose vein samples with hydrophilic interaction liquid chromatography (HILIC) was performed as previously described^28^ using an Acquity UPLC System (Waters, Ltd. Elstree, UK), coupled with LCT Premier mass spectrometer (Waters MS Technologies, Ltd., Manchester, U.K.). An Acquity UPLC BEH HILIC column (1.7 μm, 2.1 × 100 mm, Waters, USA) was used and maintained at 35 °C. Details of instruments settings (for organic and HILIC UPLC-MS analysis are provided in supplementary information section)^28^.

For UPLC-MS, data were processed using the XCMS package^30^ (version 1.46.0) in the R programing language (version 3.2.0). After peak-picking, retention time correction and grouping of the chromatographic peaks, an algorithm was used for filling zero values using the adjacent background intensity. The three-dimensional table produced consisted of features characterized by their m/z, retention time and signal intensity. The dataset was then subjected to total area normalization.

Metabolite structural assignments for UPLC-MS were conducted by: 1) matching accurate mass measurements to theoretical values from on-line databases including METLIN (http://metlin.scripps.edu/metabolites), HMDB (http://www.hmdb.ca) and LIPID MAPS, (http://www.lipidmaps.org), 2) isotopic patterns, 3) in-house developed libraries of standards^28^ and 4) MSE and/or MS/MS spectra, by matching to tandem MS experiments from online databases.

### Multivariate statistical analysis

SIMCA-P+ 12.5 statistical software (UMETRICS™, Sweden) was used to analyse the data acquired from UPLC-MS, whereas the NMR data was analysed using MATLAB R2009b (Mathworks™, 2009) with scripts developed in-house. Both principal components analysis (PCA) and orthogonal projection to latent structures discriminant analysis (OPLS-DA) were performed. The validity of the OPLS models were assessed by performing 1000 permutations with randomized scrambling of class labels. OPLS loadings S-plots derived from the OPLS-DA models were used to discern the variables driving group differences. Further univariate analysis of variance (one-way ANOVA) was performed on the complete set of data to identify the individual effects of each variable; Benjamini-Yekutieli correction was used to adjust the significance statistics to account for multiple testing^31^. Only the biomarkers which were identified by both the validated multivariate OPLS-DA models and adjusted univariate statistics were considered to be significant overall.

A multivariate partial least square (PLS) regression modelling approach was used to identify any influences of demographic and clinical confounders (Table 2) on the both aqueous NMR and non-polar UPLC-MS metabolic vein tissue profiles. For each factor (age, sex, diabetes, etc.) a PLS model was constructed whereby the y matrix was replaced with the factor under investigation instead of the sample class. Q^2^Y, R^2^X values and p values (relating to the predictive variability, variance explained and significance of the model respectively) for each model were calculated.

### miRNA analysis

A subset of tissue samples (8 test subjects and 8 controls) were analysed for microRNA expression, by Miltenyi Biotec (Bergisch Gladbach, Germany). These were chosen randomly from the cohort of 115 samples prepared for metabonomic analysis. Samples underwent RNA extraction using TRIzol (Invitrogen) under RNase-free conditions. Details of experimental protocol are given in supplementary information section. The quantity and quality of the RNA extraction was investigated by bioanalyzer and the generation of an RNA Integrity Number (RIN). A RIN value of greater than 6 was considered to be acceptable and based on this six samples (3 test and 3 control) of the original 16 samples were rejected. Electropherograms of the accepted samples indicated that there was good signal intensity in each of the sample runs. The fold change ratios of expression were calculated based on light intensity (light units) after hybridisation of the extracted RNA onto microarrays.

Functional analysis of the identified miRNA targets was performed using computational methods. Initial confirmation of miRNA nomenclature was achieved using miRNA database mirBase (www.mirbase.org), after which the miRNAs were ranked according to experimental versus control expression ratios. The 10 most correlated and the 10 most anti-correlated miRNAs were then examined for their expected regulated cellular processes using the DIANALAB database, which provided a list of potential KEGG pathways, ranked by statistical confidence. Prediction modelling tool TargetScan was then used to produce a list of mRNAs under the regulation each of the target miRNAs, which was subsequently imported into the PANTHER classification system to produce a list of pathways containing the mRNAs in question. These pathways were analysed using a statistical overrepresentation analysis to produce a p-value to identify which ones were statistical significant for the experiment. All processes that were statistically significant were incorporated into Ingenuity Pathway Analysis (IPA, http://www.ingenuity.com/products/ipa) to create a network biology system for the interaction between both the miRNAs and the metabolites identified from the metabonomic analysis. A Pearson correlation matrix was constructed to identify which miRNA expression levels correlated with concentrations of the previously identified significant metabolites. Finally, the results from all the above analyses were combined into a single IPA pathway mapping analysis, which mapped the biological functions of the differential metabolites and miRNAs.

## Electronic supplementary material

SI
