# Supplementary material for: A comprehensive characterisation of the metabolic profile of varicose veins; implications in elaborating plausible cellular pathways for disease pathogenesis
Source: Sci Rep. 2017 Jun 7;7:2989. doi: 10.1038/s41598-017-02529-y (PMC5462754; doi:10.1038/s41598-017-02529-y)
Supplement: Supplementary file 1 — SI [file 41598_2017_2529_MOESM1_ESM.pdf]

Supplementary Information

A comprehensive characterisation of the metabolic profile of varicose veins; implications in elaborating plausible cellular pathways for disease pathogenesis

Dr Muzaffar A Anwar[1] MBBS, MRCS, PhD*

Dr KN Adesina-Georgiadis [1] MSc, PhD*

Dr K Spagou[2] PhD

Dr PA Vorkas[2] MSc, PhD

Dr J Li[2] PhD

Dr Joseph Shalhoub[1] MBBS, FRCS, PhD

Professor Elaine Holmes[2] PhD

Professor Alun H Davies[1] MA, DM, DSc, FRCS

[1]Academic Section of Vascular Surgery & [2]Section of Computational and Systems Medicine

Department of Surgery and Cancer

Imperial College London, UK

*These authors share joint first authorship.

Extraction of Metabolites

Organic extraction was performed by adding 1.5 mls of MTBE/methanol (3:1) in each 2ml microtube (VWR, UK) containing tissue sample and 1mm zirconium beads (BioSpec USA). Two extraction blanks samples were also prepared for each group and run parallel with other tissue samples in order to assess if there was any metabolite carryover from previous samples and semi-quantify the presence of impurities and contaminants relevant to the extraction procedure. Samples were loaded onto a bead beater (Precellys 24, Bertin Technologies) and a homogenization cycle, consisting of 40 s shaking at 6500 Hz followed by 5 min cooling on dry ice, was repeated 4 times to maximize dissolution of the powder. Samples were centrifuged (Eppendorf 5417R) at 17949 x *g* for 20 min at 4 °C and the supernatant was taken into an Eppendorf tube. Content from each beading tube was then transferred into 5 glass vials with PTFE seal (Fisher, UK) each containing 200 µl. Glass vials containing organic metabolites were dried overnight in a fume hood at room temperature and then stored in -40°C freezer. For aqueous extraction, 1.5 mls of water/ methanol (1:1) was added in each 2ml microtube containing the sample which was then run on bead beater for 2 cycles at 6500 Hz each lasting 40 s. This was followed by Centrifuged (Eppendorf 5417R) at 17949 x *g* for 20 min at 4 °C. A total of 1.25 mL of supernatant was obtained from each sample and further divided into 5 x 250 µL aliquots. Samples were dried in a speed vacuum for 10 hours at 30 °C and stored at -40 °C pending NMR and UPLC-MS analysis.

Preparation of samples for ^{1}H-NMR spectroscopic analysis of aqueous extracts

Two aliquots of the dried aqueous extracts were combined after sequential reconstitution in 650 µL sodium phosphate buffer solution (0.2 M, 0.05% of sodium 3-trimethylsilyl-1-[2,2,3,3,-$^{2}H_4$] propionate (TSP), 70% D_2O, pH 7.4). The aliquots were vortexed for 1 min, sonicated for 5 min and vortexed for additional 1 min, followed by centrifugation for 30 s at 17945 x *g* at 4 ^{0}C. The supernatant was transferred to the second aliquot and the reconstitution procedure was repeated. The supernatant (500 uL) was then transferred into an NMR tube with an outer diameter of 5 mm.

Preparation of samples for UPLC-MS analysis of aqueous and organic extracts

Dried aqueous extracts were reconstituted in 200 µL of acetonitrile/water (95:5) for UPLC-MS hydrophilic interaction liquid chromatography (HILIC) analysis. Samples were vortexed for 1 min, sonicated for 5 min, vortexed again for 1 min and then centrifuged at 17949 x *g* at 4 °C for 8 min. Contents were then transferred into LC-MS grade glass total recovery vials (Waters, USA). A total of 50 µl from each sample was added together to make a quality control (QC) sample.

Dried organic extracts were reconstituted in 250 µL of ISP/acetonitrile/water (2:1:1). Samples were vortexed for 1 min, sonicated for 5 min, vortexed again for 1 min and then centrifuged at 17949 x *g* at 4 °C for 8 min. Following centrifugation at 17949 x *g* at 4 °C for 8 min, contents were transferred into LC-MS grade glass vials with inserts (Waters, USA). 200 µl of each sample was put inside the 1.8mm LCMS glass vials and the remaining 50 µl of each sample was added together to form the quality control (QC) sample.

Instrument Settings

HILIC-UPLC-MS: The composition of the mobile phases was: 0.1% (v/v) formic acid (FA) and 10 mM ammonium acetate in acetonitrile/H_2O (95:5) (A), and 0.1% (v/v) FA and 10 mM ammonium acetate in acetonitrile/H_2O (50:50) (B). The source temperature was set at 120 ^oC and desolvation gas temperature at 400 ^oC. The electrospray ionization (ESI) conditions for aqueous analysis on UPLC-MS were: cone gas flow of 25 L/hr, desolvation gas flow 800 L/hr, capillary voltage 3000 V for ESI positive (ESI+) and 2000 V for ESI negative (ESI-) modes, and cone voltage 25 V. The instrument was set to acquire in a mass-to-charge ratio (m/z) range of 50-1000 with scan time of 0.2 s and inter-scan delay of 0.01 s.

Organic extracts-RP-UPLC-MS: Mobile phases consisted of acetonitrile/water (60:40) with 10 mM ammonium formate and 0.1%FA (A), and ISP/acetonitrile (90:10) with 10 mM ammonium formate and 0.1% FA. ESI conditions were set with the source temperature 120 ^oC, desolvation temperature 400 ^oC, cone gas flow 25 L/hr, desolvation gas flow 800 L/hr, capillary voltage 3000 V for ESI+ and 2500 V for ESI-, and cone voltage of 30 V. The instrument was set to acquire over the m/z range 50-1200 in V mode with scan time of 0.2 s and an inter-scan delay of 0.02 s.

For all UPLC-MS experiments, samples were maintained at 4 ^oC during analyses. Leucine enkephalin (200 pg/µL, in acetonitrile/water 50:50, 0.1% FA) was used as for lock mass correction with double scan acquisition every 30s. The instrument was calibrated before analyses using 0.5mM sodium formate solution.The gradient programs for all UPLC-MS analyses are provided in Supplementary Table 4.

miRNA analysis

Samples were snap frozen and powdered, and subsequently 1ml of TRIzol was added per 100mg of tissue and left to solubulize at room temperature. 0.2ml of chloroform was added per 100mg of tissue and vortexed for 15 seconds to enhance phase separation. Samples were then centrifuged at 10000G for 10 minutes and the supernatant collected and 0.5 ml of isopropanol added to precipitate the RNA. The samples were centrifuged again at 10000G

for 10minutes and the supernatant discarded. The pellet was re-suspended in (1x) sodium dodecyl sulphate buffer and NaOAc was added to 3 M. The RNA samples, which were quality-checked via the Agilent 2100 Bioanalyzer platform (Agilent Technologies). Once RNA quality was assured, 100ng of each of the samples was labelled and hybridised by incubating for 20 hours at 55 ^{o}C to a Agilent Human microRNA Microarrays 8x60K v16 (Agilent Technologies), after which the microarrays were washed once with the Agilent Gene Expression Wash Buffer for 5 min at room temperature followed by a second wash with preheated Agilent Gene Expression Wash Buffer 2 (37 °C) for 5 min. The fluorescence signals of the hybridized Agilent Microarrays were detected using Agilent's Microarray Scanner System which determines feature intensities and allows for the possibility to compare two single intensity profiles in a ratio experiment.

Pathway analysis nodes elaboration

APOA1	Apolipoprotein A-1
SST	Somatostatin
F2	Coagulation factor II Thrombin is a protein coding gene
BMP1	Bone morphogenetic protein 1
TAC1	Preprotachykinin-1
IFNG	Interferon gamma
CCK	Cholecystokinin
SSTR5	Somatostatin receptor 5
ATP4A	Adenosine triphosphate H^+/K^+ transporting alpha subunit
ATP4B	Adenosine triphosphate H^+/K^+ transporting beta subunit
ADRB3	Adrenoceptor beta 3
STX3	Syntaxin 3 protein coding gene
BMP1	Bone morphogenetic protein 1
GRIA2	Glutamate ionotropic receptor alpha-amino-3-hydroxy-5-methyl-4-isoxazolepropionate (AMPA) type subunit 2 protein coding gene
GRIA3	Glutamate ionotropic receptor alpha-amino-3-hydroxy-5-methyl-4-isoxazole propionate (AMPA) type subunit 3 protein coding gene
GRIA1	Glutamate ionotropic receptor alpha-amino-3-hydroxy-5-methyl-4-isoxazole propionate (AMPA) type subunit 1 protein coding gene
INSR	Insulin receptor

AGTR1	Angiotensin II Receptor Type 1
ADRB2	Adrenoceptor beta 2
GNA1	Glucosamine-Phosphate N-Acetyltransferase 1
ADRA1A	Adrenoceptor Alpha 1A
NMDA Receptor	*N*-methyl-D-aspartate receptor
APP	Amyloid Beta Precursor Protein
ADRA1B	Adrenoceptor Alpha 1B
ADRB1	Adrenoceptor beta 1
SYN2	Synapsin II
ADRA1D	Adrenoceptor Alpha 1D
APPBP2	Amyloid Beta Precursor Protein Binding Protein 2
ADRBK1	Adrenergic Beta Receptor Kinase 1
SORL1	Sortilin Related Receptor 1
ARHGEF12	Rho Guanine Nucleotide Exchange Factor 12
TRIM54	Tripartite Motif Containing 54
USP33	Ubiquitin Specific Peptidase 33
PTEN	Phosphatase And Tensin Homolog
MAP1B	Microtubule Associated Protein 1B
INA	Internexin Neuronal Intermediate Filament Protein Alpha
HMGCR	3-Hydroxy-3-Methylglutaryl-CoA Reductase
CREM	CAMP Responsive Element Modulator
RB1	RB Transcriptional Corepressor 1
CBL	Cbl Proto-Oncogene

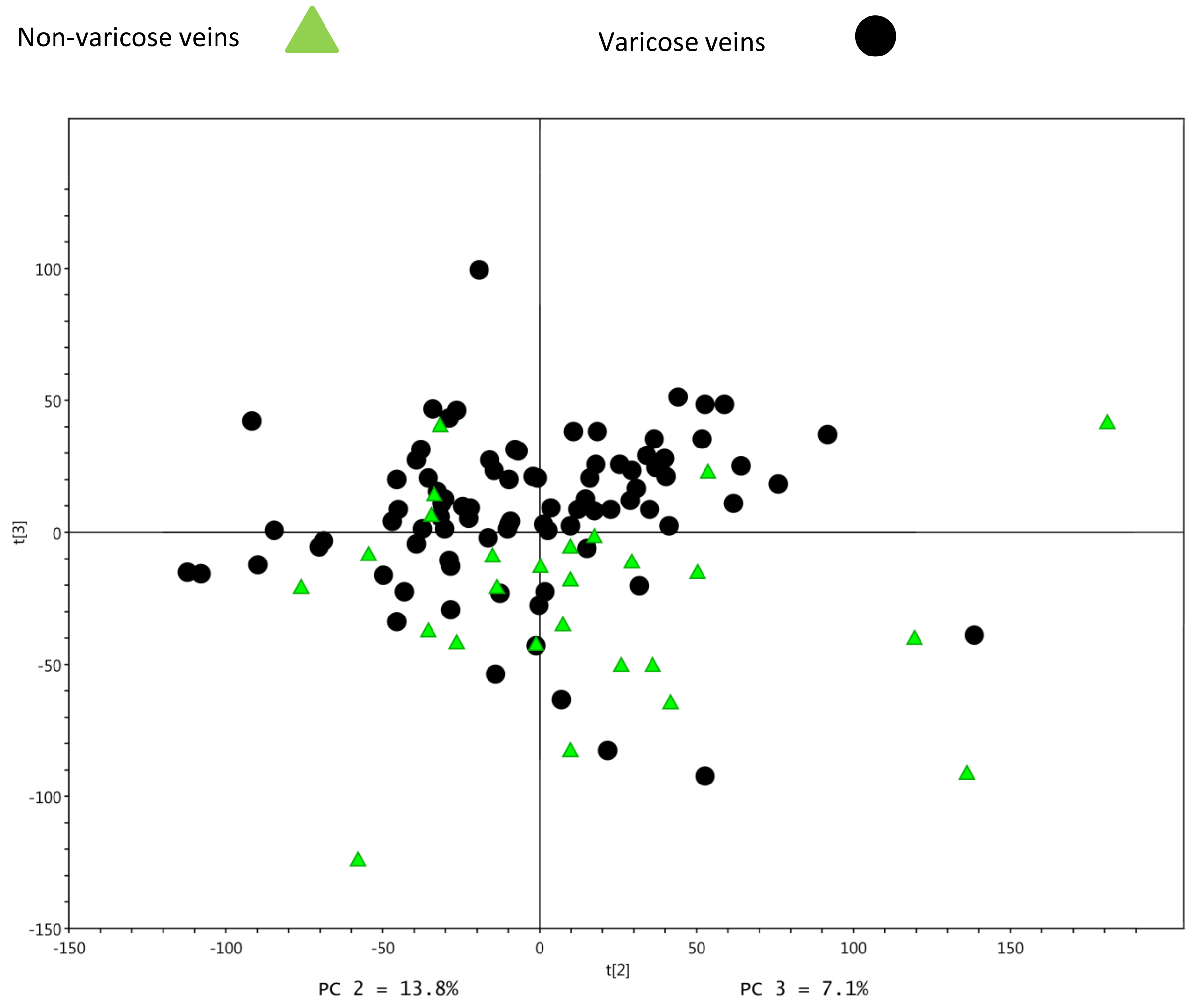

Supplementary Figure 1. PCA score plots of data acquired from of ^{1}H NMR spectroscopic analysis of aqueous extracts showing the overall variation in the samples.

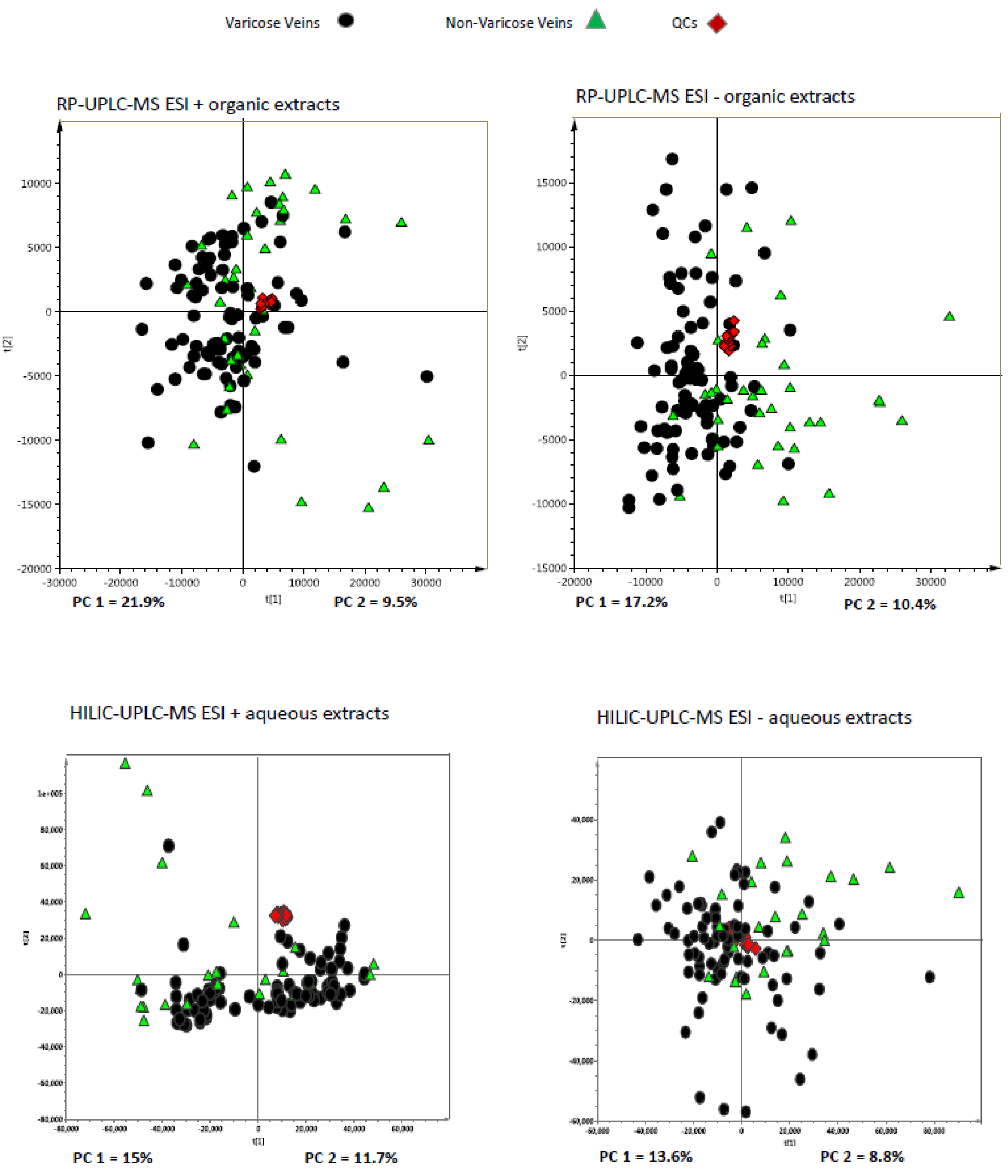

Supplementary Figure 2. PCA score plots of data acquired from UPLC-MS organic and HILIC ESI +/- showing the overall variation in the samples and instrument stability (good clustering of QCs samples).

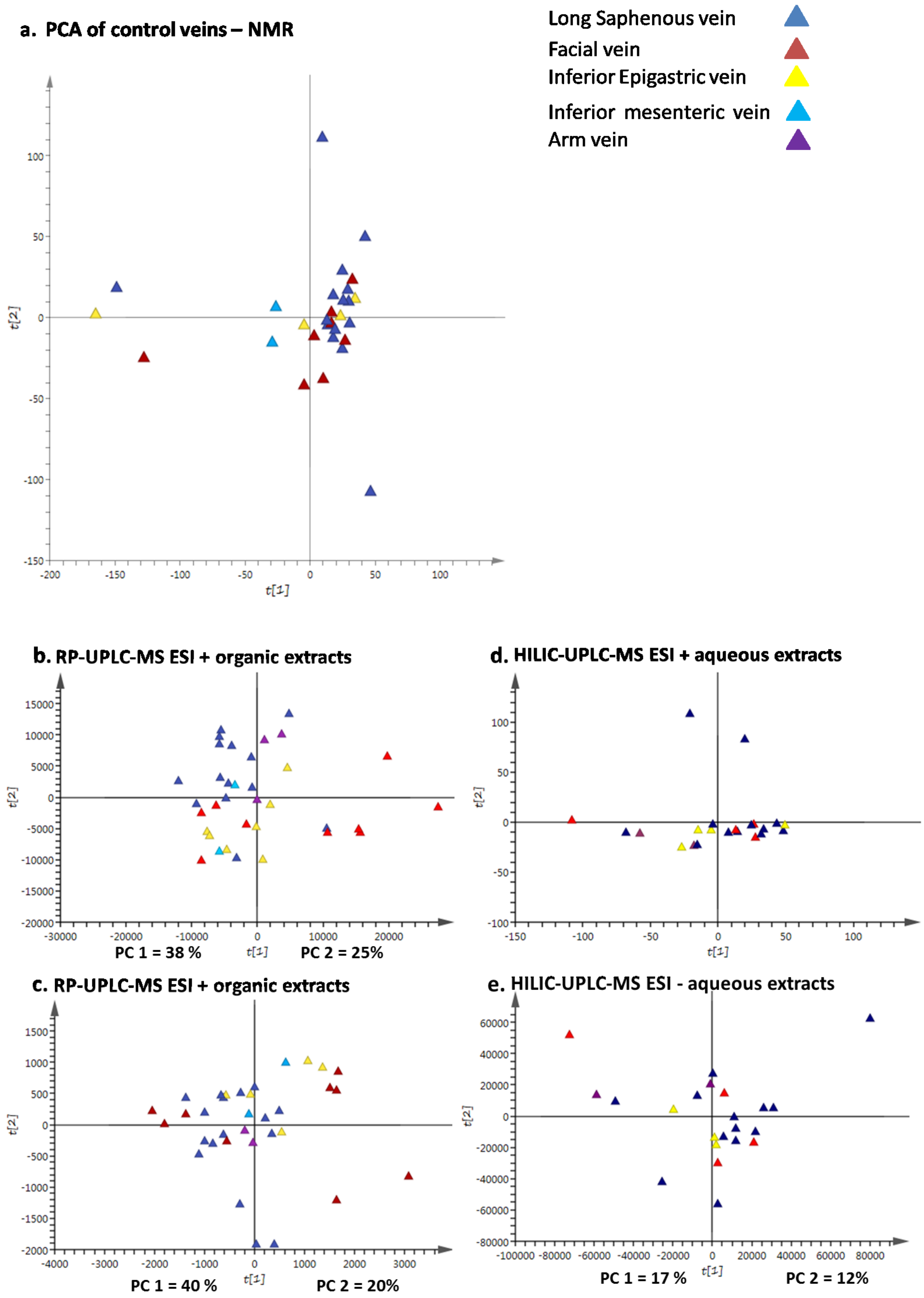

Supplementary Figure 3. PCA scores plots of control veins samples only, coloured according to anatomical origin of the control sample. Each panel represents one of the five analyses performed (as labelled).

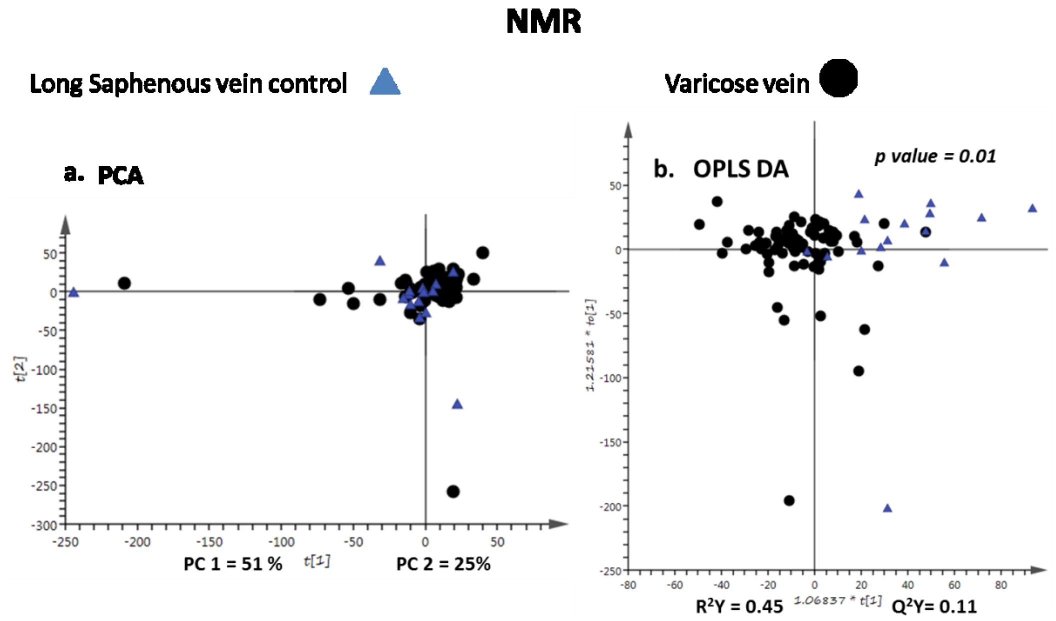

Supplementary Figure 4. PCA (a) and OPLS-DA (b) scores plot of models comparing NMR data from only long saphenous vein controls and varicose veins samples.

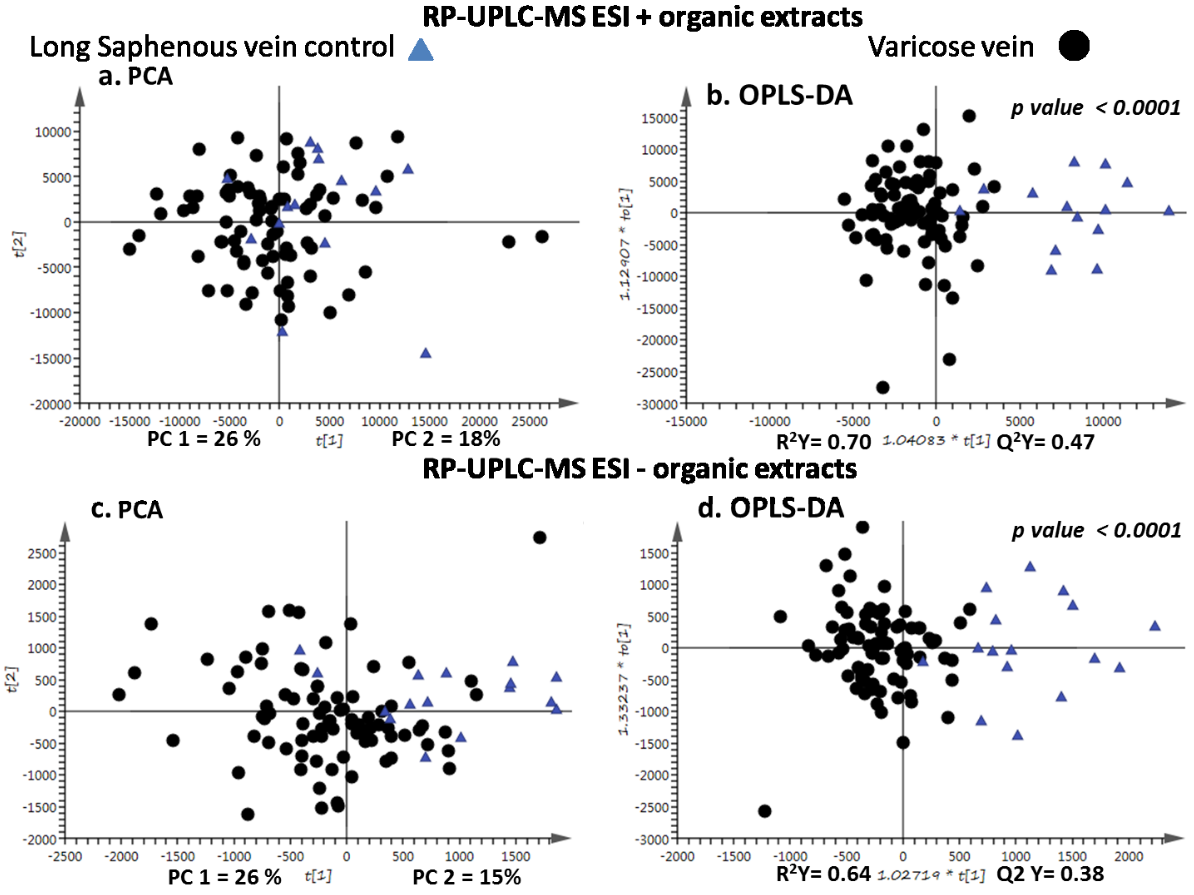

Supplementary Figure 5. PCA (a and c) and OPLS-DA (b and d)scores plots of models comparing UPLC-MS lipid profiling data from only long saphenous vein controls and varicose veins samples. (a) and (b) are from the ESI+ while (c) and (d) are from the ESI- data.

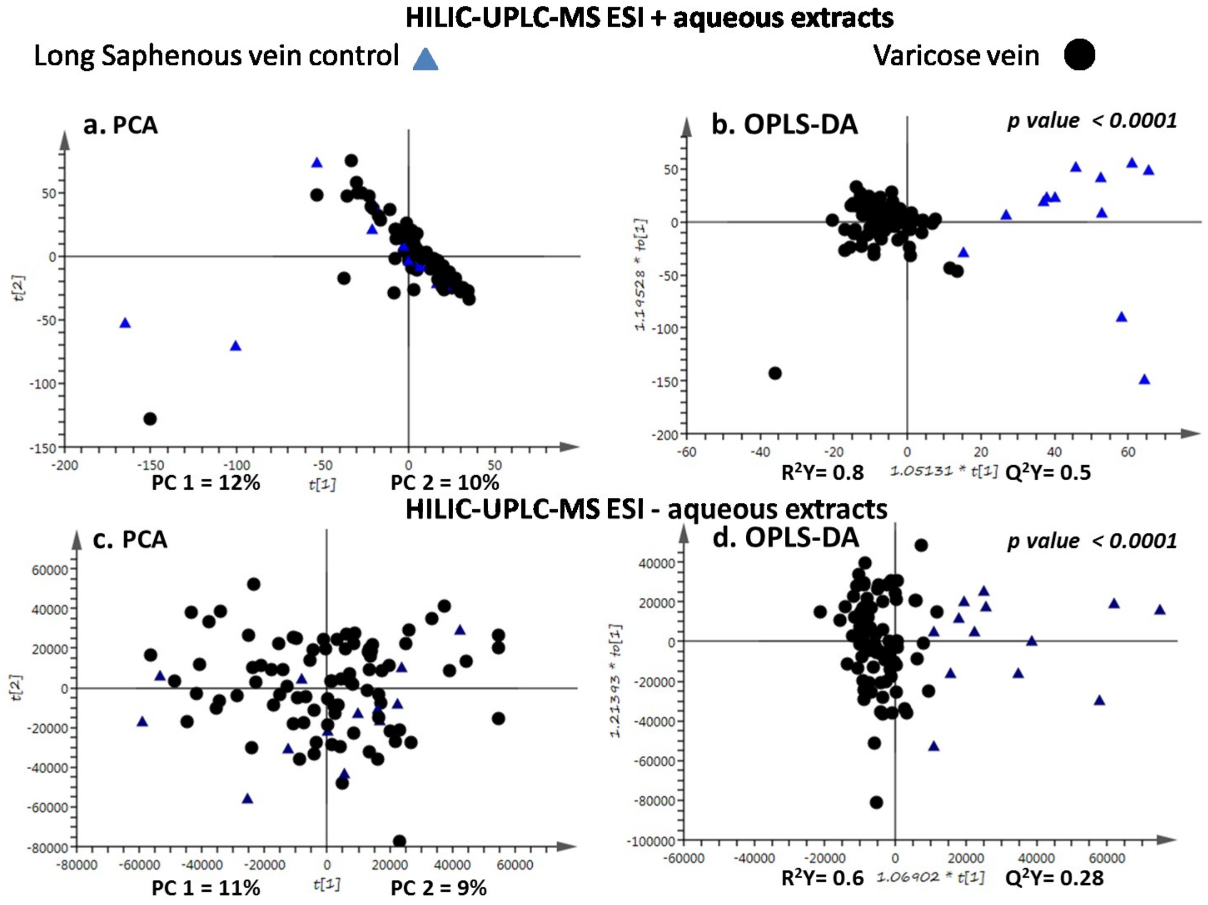

Supplementary Figure 6. PCA (a and c) and OPLS-DA (b and d)scores plots of models comparing UPLC-MS HILIC profiling data from only long saphenous vein controls and varicose veins samples. (a) and (b) are from the ESI+ while (c) and (d) are from the ESI- data.

Supplementary Table 1. Details of unknown differential features identified between varicose and non-varicose veins groups on UP-LCMS ESI +/- analysis.

Met Name	Mol Formula as Detected	Retention Time (min)	m/z (found)	m/z (theor)	Δppm	p-value	Fold change	Higher in group
UKN(0878.7034_13.17-LP-POS)	n/a	13.17	0878.7034	n/a	n/a	0.015	-1.3	Non-VVs
UKN(0603.5357_05.97-LP-POS)	n/a	05.97	603.5357	n/a	n/a	0.0008	1.4	VVs
UKN(0764.5247_05.78-LP-POS)	n/a	05.78	764.5247	n/a	n/a	0.003	1.3	VVs
UKN(0769.5362_08.13-LP-POS)	n/a	08.13	0769.5362	n/a	n/a	0.016	1.3	VVs
UKN(0792.5613_07.64-LP-POS)	n/a	07.64	0792.5613	n/a	n/a	0.001	1.3	VVs
UKN(0809.6107_08.01-LP-POS)	n/a	08.01	0809.6107	n/a	n/a	0.0009	1.3	VVs
UKN(0809.6470_07.79-LP-POS)	n/a	07.79	0809.6470	n/a	n/a	< 0.0001	1.6	VVs
UKN(0837.6761_10.28-LP-POS)	n/a	10.28	0837.6761	n/a	n/a	< 0.0001	2.3	VVs
UKN(0522.2838_01.01-LP-NEG)	n/a	01.01	0522.2838	n/a	n/a	0.008	-1.5	Non-VVs
UKN(0680.6206_13.75-LP-NEG)	n/a	13.75	0680.6206	n/a	n/a	0.007	-1.5	Non-VVs
UKN(0703.5616_13.55-LP-NEG)	n/a	13.55	0703.5683	n/a	n/a	0.009	-1.3	Non-VVs
UKN(0166.0481_00.60-LP-NEG)	n/a	00.60	0166.0481	n/a	n/a	0.0007	-1.7	Non-VVs
UKN(0788.5452_07.73-LP-NEG)	n/a	07.73	0788.5452	n/a	n/a	0.001	1.3	VVs
UKN(0790.5477_07.68-LP-NEG)	n/a	07.68	0790.5477	n/a	n/a	0.0004	1.3	VVs
UKN(0794.5723_10.08-LP-NEG)	n/a	10.08	0794.5723	n/a	n/a	0.007	1.2	VVs
UKN(0882.6253_09.36-LP-NEG)	n/a	09.36	0882.6253	n/a	n/a	0.0005	1.5	VVs
UKN(0913.5854_07.01-LP-NEG)	n/a	07.01	0913.5854	n/a	n/a	<0.0001	1.7	VVs

Supplementary Table 2. The association of patient demographic variables with non-polar metabolic profile acquired from analysis of veins tissue extracts on NMR and UP-LCMS ESI +.

*PLS-DA model for the first component

NMR			
	R^2Y	Q^2Y	P value
Age	0.037	-0.006	1
Sex	0.185	-0.167	1
Smoke	0.237	-0.081	1
HTN	0.288	-0.020	1
Diabetes	0.461	-0.067	1
PVD	0.455	-0.175	1
IHD	0.455	-0.003	1
Stroke	0.319	-0.111	1
Cancer	0.287	-0.194	1
HC	0.291	-0.105	1
Aspirin	0.311	0.015	0.69
Statins	0.317	-0.051	1
Lisin	0.324	0.068	0.12
Amlodipine	0.195	-0.080	1
Deuretics	0.242	-0.128	1
UPLC MS organic ESI +			
	R^2Y	Q^2Y	P value
Age	0.271	0.052	0.6
Sex	0.102	-0.254	1
Smoke	0.190	-0.219	1
HTN	0.214	-0.034	1
Diabetes	0.457	0.047	0.81
PVD	0.216	0.015	0.42
IHD	0.190	-0.142	1
Stroke	0.245	-0.129	1
Cancer	0.247	-0.178	1
HC	0.239	-0.138	1
Aspirin	0.317	-0.052	1
Statins	0.185	-0.019	1
Lisin	0.220	0.051	0.51
Amlodipine	0.216	-0.111	1
Deuretics	0.223	-0.033	1
UPLC MS organic ESI -			
	R^2Y	Q^2Y	P value
Age	0.271	0.052	0.6
Sex	0.198	0.012	0.49
Smoke	0.226	-0.056	1
HTN*	0.207	0.053	0.05
Diabetes	0.177	-0.1	1
PVD	0.24	-0.01	1
IHD	0.218	-0.099	1
Stroke	0.166	-0.006	1
Cancer	0.067	-0.073	1
HC	0.15	-0.1	1

Aspirin	0.196	-0.002	1
Statins	0.255	-0.1	1
Lisin	0.267	-0.010	1
Amlodipine	0.167	-0.088	1
Deuretics	0.177	-0.041	1
UPLC MS HILIC ESI +			
	R^2Y	Q^2Y	P value
Age	0.469	0.025	0.39
Sex	0.492	-0.121	1
Smoke	0.431	-0.146	1
HTN	0.484	0.064	0.19
Diabetes	0.561	-0.10	1
PVD	0.446	-0.034	1
IHD	0.516	-0.108	1
Stroke	0.363	-0.161	1
Cancer	0.392	-0.21	1
HC	0.282	-0.21	1
Aspirin	0.404	-0.21	1
Statins	0.489	-0.185	1
Lisin	0.551	0.028	0.25
Amlodipine	0.194	-0.074	1
Deuretics	0.534	-0.098	1
OPLS MS HILIC ESI -			
	R^2Y	Q^2Y	P value
Age	0.046	-0.049	1
Sex	0.468	-0.154	1
Smoke	0.321	-0.152	1
HTN	0.495	0.043	0.09
Diabetes	0.562	-0.132	1
PVD	0.247	0.013	0.49
IHD	0.468	-0.154	1
Stroke	0.429	-0.21	1
Cancer	0.375	-0.21	1
HC	0.202	-0.1	1
Aspirin	0.45	0.049	0.73
Statins	0.468	-0.014	0.54
Lisin	0.512	0.018	0.05
Amlodipine	0.442	-0.153	1
Deuretics	0.508	-0.146	1

Supplementary Table 3. Gradient programs of chromatography of HILIC-UPLC-MS of aqueous and RP-UPLC-MS profiling of organic extracts.

HILIC-UPLC-MS

Time (min)	Flow rate ml/ min	% A	% B	curve
Initial	0.400	99.0	1.0	-
2.00	0.400	99.0	1.0	6
8.00	0.400	45.0	55.0	6
9.00	0.400	1.0	99.0	6
9.10	0.600	1.0	99.0	6
11.00	0.600	99.0	1.0	6
11.10	0.600	99.0	1.0	6
19.00	0.600	99.0	1.0	6
19.10	0.400	99.0	1.0	6
23.00	0.400	99.0	1.0	6

Organic extracts-RP-UPLC-MS

Time (min)	Flow rate	% A	% B	curve
Initial	0.400	60.00	40.00	-
2.00	0.400	57.00	43.00	6
2.10	0.400	50.00	50.00	1
12.00	0.400	46.00	54.00	6
12.10	0.400	30.00	70.00	1
18.00	0.400	1.00	99.9	6
18.10	0.400	60.00	40.00	6
20.0	0.400	60.00	40.00	-
